# Insulin-like growth factor-1 enhances neuroprotective effects of neural stem cell exosomes after spinal cord injury via an miR-219a-2-3p/YY1 mechanism

**DOI:** 10.18632/aging.102568

**Published:** 2019-12-17

**Authors:** Ke Ma, Huiyou Xu, Jian Zhang, Fei Zhao, Haiqian Liang, Hongtao Sun, Ping Li, Sai Zhang, Renjie Wang, Xuyi Chen

**Affiliations:** 1 Department of Neurosurgery, Characteristic Medical Center of Chinese People’s Armed Police Force, Institution of Brain Trauma and Neurology Disease of People's Armed Police Forces, Tianjin Key Laboratory of Neurotrauma Repair, Tianjin 300162, China

**Keywords:** spinal cord injury (SCI), microRNAs (miRNAs), exosomes, apoptosis

## Abstract

Spinal cord injury (SCI) remains the most common cause of paralysis, and there are no effective therapies for SCI patients. Neural stem cell (NSC)-derived exosomes can attenuate apoptosis and neuroinflammation after traumatic spinal cord injury, but the mechanisms underlying these effects remain unclear. Here, we examined the efficacy of miRNAs isolated from exosomes as treatments for SCI and characterized their mechanisms of action. Furthermore, we evaluated the effects of exosomes formed in the presence of insulin growth factor-1 (IFG-1, IGF-Exo), which promotes neural proliferation and regeneration, as well as normal exosomes (Nor-Exo) and compared control and H_2_O_2_-treated groups both *in* *vitro* and *in* *vivo*. Using microRNA sequencing and qRT-PCR, we identified miR-219a-2-3p, levels of which were higher in the IGF-Exo than Nor-Exo group and played crucial anti-inflammatory and anti-apoptosis roles. Additional experiments revealed that IGF-Exo inhibits YY1 expression through up-regulation of miR-219a-2-3p. This in turn inhibits the NF-κB pathway, partly inhibiting neuroinflammation and promoting the neuroprotective effects after SCI.

## INTRODUCTION

Spinal cord injury (SCI), which typically results from traffic accidents or long falls, is the most common cause of paralysis [1]. Two key mechanisms play roles in the pathology of SCI: a) primary injury resulting from bruising and compressive lesions, and b) secondary injuries, including inflammation, formation of glial scars, microvascular bleeding, and upregulation of apoptotic factors, all of which inhibit axonal regeneration [2, 3]. There are no effective therapies for patients with SCI, and novel treatments are urgently needed.

Exosomes are nanosized extracellular vesicles 30–100 nm diameter which are formed as a result of inward budding in multiple cell types [4]. They were first discovered in sheep reticulocytes in 1983 [5]_,_ and the term “exosome” was coined by Johnstone in 1987 [6]. Exosomes may serve as biomarkers in many diseases due to the important role they play in intercellular communication and transport of proteins, mRNAs, and miRNAs into target cells [7, 8]. Exosomes can also have therapeutic effects in the nervous and respiratory systems [9]. Other studies demonstrated that exosomes derived from mesenchymal stem cells (MSCs) had therapeutic effects in liver, cardiovascular, and kidney diseases [10–12]. Furthermore, systemic administration of exosomes derived from MSCs promoted neurovascular reshaping and functional recovery after stroke in rats [13]. Exosomes can also reduce cognitive impairments after traumatic brain injury [14]. Recent research has demonstrated that exosomes containing large amounts of miRNA are pivotal for intercellular communication in the nervous system [15]. The miRNAs contained in MSC-derived exosomes also play important roles in intercellular signaling more generally [16]. Current knowledge indicates that the process by which miRNAs are loaded into exosomes is not random and differs in different cell types [17]. In addition, a recent study suggests that exosomes facilitate the transfer of miR-155 from smooth muscle cells to endothelial cells, which induces endothelial injury and promotes atherosclerosis [18].

Although neural stem cell-derived exosomes attenuate apoptosis and neuroinflammation after traumatic spinal cord injury [19], the mechanism underlying this effect remains unknown. Because insulin-like growth factor-1 (IGF-1) promotes regeneration after neural injury and proliferation in neural cells [20–22], we hypothesized that it may enhance the protective and regenerative effects of NSC-derived exosomes after SCI.

The pathological effects of SCI are primarily a result of neuroinflammation, apoptosis, and oxidative stress injury [23–25]. We therefore established an H_2_O_2_-induced PC12 cell injury model to mimic SCI *in* *vitro* [26] and a rat SCI model using Allen’s modified appliance *in* *vivo* [27, 28]. In this study, we examined the effects of IGF-1-induced (IGF-Exo) and normal exosomes (Nor-Exo) on neural inflammation, apoptosis, and regeneration after SCI and identified an miRNA-dependent mechanism responsible for those effects.

## RESULTS

### Preparation and characterization of IGF-Exo

**Figure 1:**
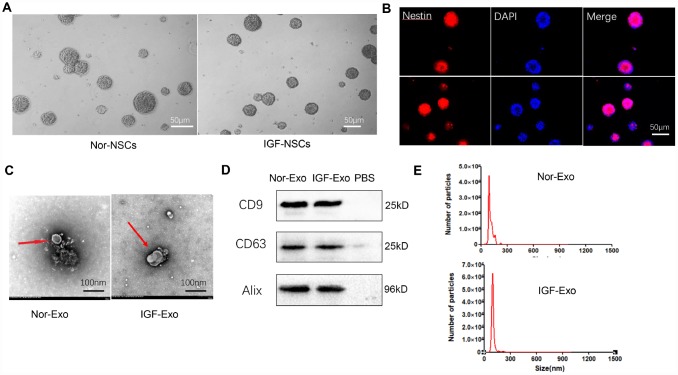
**Characteristics of neural stem cells (NSCs) and exosomes derived from NSCs.** (**A**) Morphology of neurospheres with typical shape examined by light microscopy. (**B**) Nestin immunofluorescence (red), a marker of NSCs, in neurospheres. (**C**) Exosome morphology examined by transmission electron microscopy. (**D**) Western blot analysis of exosome surface markers. (**E**) Particle size distribution of Nor-Exo and IGF-Exo by Nano Sight.

Neural stem cells were obtained from E15 fetal rat cerebral cortex and cultured in medium until neurospheres of similar sizes and shapes formed **(**Fig. 1A). These neurospheres were immunopositive for the NSC marker nestin (shown in red) (Figure 1B). Rat NSCs collected from passage 3 were cultured in two kinds of culture medium: complete medium (DMEM/F12 medium supplemented with 20 ng/mL EGF, 10 ng/mL bFGF, 1× B27 supplement, 100 U/mL streptomycin, and 100 U/mL penicillin) or IGF-1 medium (100 ng/μL IGF-1 in complete medium). Exosomes were isolated from cell supernatants by ultracentrifugation. The shapes and sizes of both types of NSC-derived Exo were examined using transmission electron microscopy. While both the normal (Nor-Exo) and IGF-Exo had diameters of 30–300 nm, and the mean diameter of the IGF-Exo was slightly larger than that of the Nor-Exo (Figure 1C). Western blot analysis indicated that levels of the exosomal markers CD9, CD63, and Alix were high in both Nor-Exo and IGF-Exo (Figure 1D). Nanosight analysis of Exo size distributions revealed that the mean diameters of Nor-Exo and IGF-Exo were 101.1 ± 19.0 nm and 139.3 ± 34.0 nm, respectively (Figure 1E).

### IGF-Exo inhibits apoptosis and promotes regeneration in neural cells

**Figure 2:**
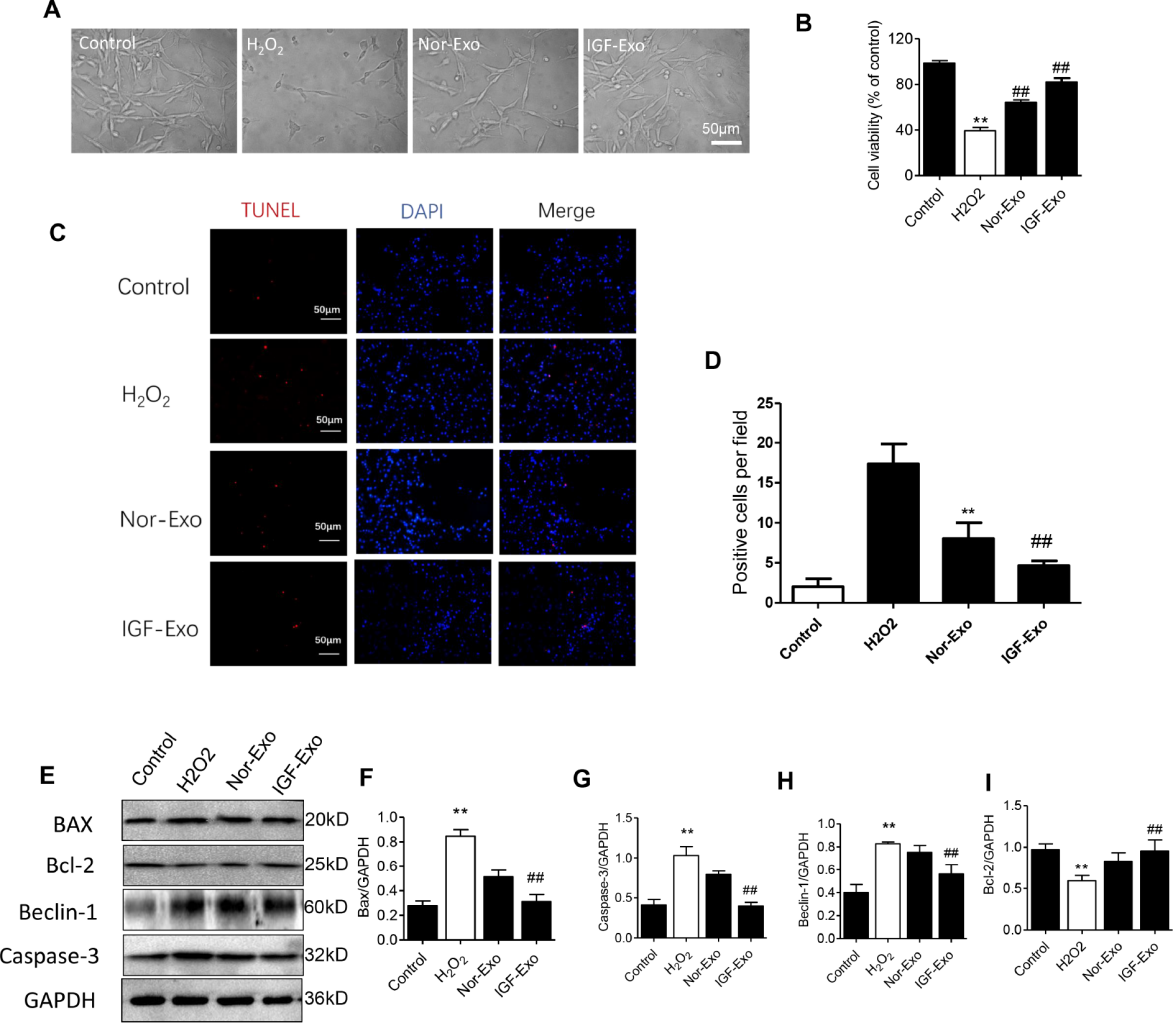
**IGF-Exo inhibited H_2_O_2_-induced neural apoptosis in PC12 cells *invitro*.** (**A**) Morphology for each experimental group examined by light microscopy (control group: PC12 cells without treatment; H_2_O_2_ group: PC12 cells treated with H_2_O_2_ for 24h; Nor-Exo group: PC12 cells pretreated with Nor-Exo for 24h followed by H_2_O_2_ for 24h; IGF-Exo group: PC12 cells pretreated with IGF-Exo for 24h followed by H_2_O_2_ for 24h). (**B**) Cell viability in each experimental group; (**C**) TUNEL staining (red) indicating cell apoptosis in each experimental group; nuclear DAPI stain in blue. (**D**) TUNEL-positive cell numbers per field for each experimental group. (**E**) Western Blot analysis of apoptotic and anti-apoptotic proteins. (**F**–**I**) Relative expression of BAX, Bcl-2, Beclin-1 and Caspase-3. Data are expressed as means ± SD (analysis of variance followed by Student-Newman-Keuls *post hoc* test). **P < 0.01 *vs*. control group; ##*P* < 0.01 *vs*. H_2_O_2_ group.

We investigated the protective effect of IGF-Exo in PC12 cells treated with H_2_O_2_ to establish a cellular model of neural injury. CCK-8 assays showed that the 50% lethal dose of H_2_O_2_ was 200 μM for 24h in PC12 cells (Supplementary Figure 1A), and the optimal dose for IGF-1 in NSC culture was 200 ng/mL for 24h (Supplementary Figure 1B). After 24h of treatment with 200 μM H_2_O_2_, PC12 cells had damaged axons and decreased in number compared with the sham group. Additionally, cell viability was higher and axons were longer in IGF-Exo group PC12 cells than in the injury model group or Nor-Exo group (*P <* 0.05) (Figure 2A, 2B). In the TUNEL immunofluorescence assay, the ratio of TUNEL-positive cells in the IGF-Exo group was much higher than in the injury model group and slightly higher than in the Nor-Exo group (*P* < 0.05) (Figure 2C, 2D). To further explore the relationship between IFG-Exo and neural cell apoptosis, we measured Bax, Bcl-2, Beclin-1, and caspase-3 levels in the four PC12 cell groups. Compared with the sham group, Bax, Beclin-1, and caspase-3 expression were increased, while Bcl-2 expression decreased, in the injury group significantly increased (*P* < 0.05). Bax, Beclin-1, and caspase-3 expression were also higher in the IGF-Exo group than in the injury group or the Nor-Exo (*P* < 0.05) (Figure 2E–2I).

### IGF-Exo reduces lesion size and promotes functional recovery after SCI

**Figure 3:**
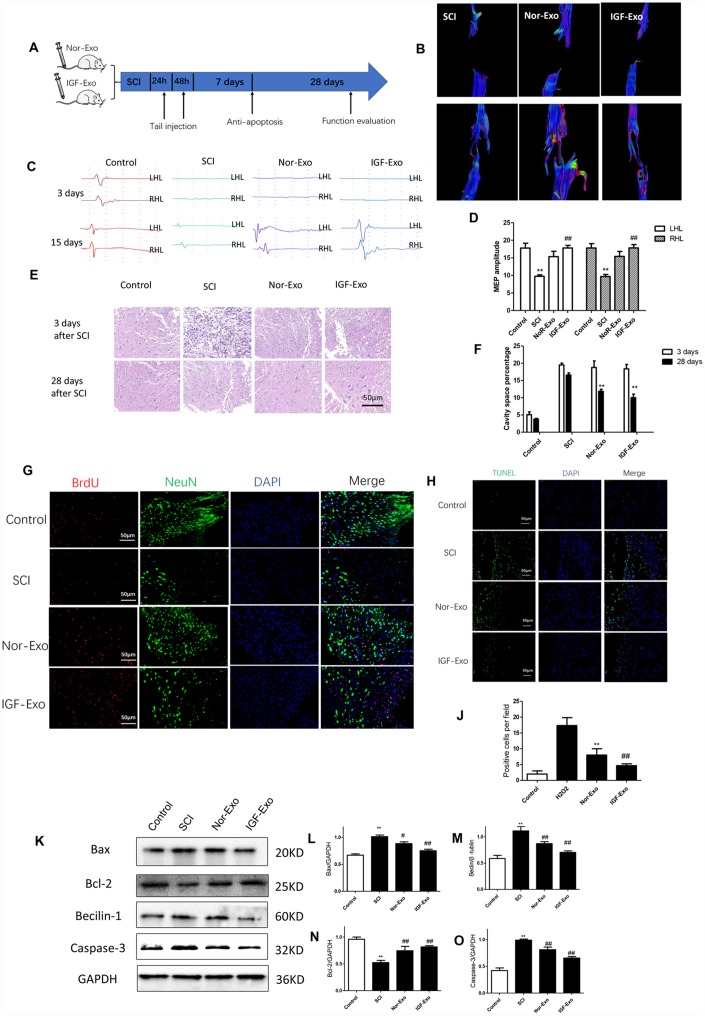
**IGF-Exo inhibited neural apoptosis and neuroinflammation after SCI *invivo*.** (**A**) Schematic of tail intravenous injections of Nor-Exo and IGF-Exo in SCI model rats. (**B**) DTIs constructed for the SCI, Nor-Exo, and IGF-Exo groups at 1 day and 28 days after surgery. (**C**) Neuroelectrophysiological examination results for each experimental group (control, SCI, Nor-Exo, and IGF-Exo) at 3 days and 28 days after surgery. (**D**) MEP amplitudes for each experimental group at 3 days and 28 days after surgery. (**E**) Hematoxylin-Eosin staining of sections containing SCI lesions in each experimental group at 3 days and 28 days after surgery. (**F**) Cavity space percentages for each experiment group at 3 days and 28 days after surgery. (**G**) BrdU and NeuN immunofluorescence indicative of neuron regeneration in each experimental group at 28 days after surgery. (**H**) TUNEL staining (green) indicative of apoptosis after SCI in each experimental group; DAPI in blue. (**J**) Numbers of TUNEL-positive cells per field for each experimental group. (**K**–**O**) Western Blot analysis of pro- and anti-apoptotic proteins (BAX, Bcl-2, Beclin-1, and Caspase-3). Data are expressed as means ± SD (analysis of variance followed by Student-Newman-Keuls *post* *hoc* test). **P < 0.01, *vs*. control group; ##P < 0.01, *vs*. SCI group.

To test the neuroprotective effect of IGF-Exo *in* *vivo*, we established a rat model of acute SCI using a modified Allen’s weight drop apparatus (Supplementary Figure 2). Rats received systemic IGF-Exo, sham, PBS, or Nor-Exo injections via the tail vein (Figure 3A). BBB locomotor scores were markedly lower in SCI group rats than in sham rats at 1, 3, 7, 14, and 28 days after SCI (*P <* 0.01). Compared with the SCI group, BBB scores were higher in Nor-Exo and IGF-Exo rats at 7, 14, and 28 days after SCI (*P* < 0.05). Moreover, the scores of IGF-Exo rats were higher than those of Nor-Exo rats at 14 and 28 days post-SCI (*P <* 0.05) (Supplementary Figure 3). MRI and neuroelectrophysiological examinations were also used to evaluate whether IGF-Exo promoted recovery from SCI recovery. DTI indicated that reconnection of the neural fasciculus was increased in the IGF-Exo group compared to the Nor-Exo and SCI groups (Figure 3B, Supplementary Figures 4, 5). Neuroelectrophysiological examination revealed that MEP amplitudes were higher in the IGF-Exo group than in the Nor-Exo and SCI groups (Figure 3C, 3D). In longitudinal sections of rat spinal cords collected for HE staining 28 days after SCI, injury areas (cavity) were smaller in the IGF-Exo group than in the SCI and Nor-Exo groups (*P <* 0.05) (Figure 3E, 3F). Immunostaining analysis of BrdU, a marker of new neurons, and NeuN, a general neural marker, revealed that BrdU and NeuN staining intensity were both lower in IGF-Exo group lesion areas than in the SCI and Nor-Exo groups at 28 days after SCI (*P <* 0.05) (Figure 3G). In a TUNEL assay performed 3 days after SCI, the TUNEL-positive cell (red) ratio was higher in the IGF-Exo group than in the injury model group and slightly higher than in the Nor-Exo group (*P* < 0.05) (Figure 3H, 3J).

Western blot analysis of Bax, Bcl-2, Beclin-1, and caspase-3 levels in the four groups of SCI rats were used to assess the anti-apoptosis effects of IGF-Exo *in* *vivo*. Compared with the sham group, Bax, Beclin-1, and caspase-3 expression increased, while Bcl-2 expression decreased, in the SCI group (*P* < 0.05). However, Bax, Beclin-1, and caspase-3 expression was significantly higher in the IGF-Exo group than in the SCI and Nor-Exo groups (*P* < 0.05) (Figure 3K–3O).

### Antiapoptotic miR-219a-2-3p is highly increased in IGF-Exo

**Figure 4:**
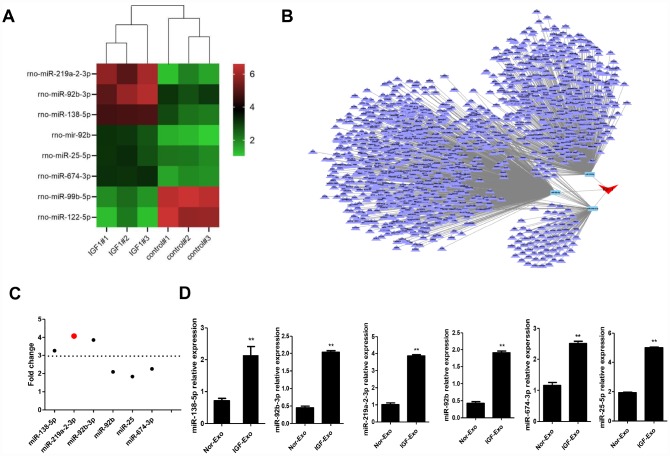
**Screening and identification of upregulated miRNAs in Nor-Exo and IGF-Exo.** (**A**) Heat map showing the 6 miRNAs for which expression was upregulated ≥ 2-fold in IGF-Exo compared to Nor-Exo. (**B**) IGF-1-induced miRNA regulation network. (**C**) Among the 6 upregulated miRNAs, miR-219a-2-3p expression increased the most. (**D**) qRT-PCR comparison of rno-miR-219a-2-3p, 138-5p, 92b-3p, 92b, 25-5p, and 674-3p expression between Nor-Exo and IGF-Exo from 3 independent experiments. *P < 0.05, **P < 0.01 for IGF-Exo *vs*. Nor-Exo.

To illuminate the mechanism underling the neuroprotective effects of IGF-Exo, we characterized exosomal miRNA content though miRNA sequencing analysis. Six miRNAs were upregulated and 2 were downregulated in IGF-Exo compared to Nor-Exo (Figure 4A–4C; Supplementary Figure 6). qRT-PCR analysis was then performed to confirm that expression was higher for the 6 upregulated miRNAs identified in the sequencing analysis. All 6 miRNAs, namely rno-miR-138-5p (P < 0.05), rno-miR-219a-2-3p (P < 0.05), rno-miR-92b-3p (P < 0.01), rno-miR-92b (P < 0.05), rno-miR-25-5p (P < 0.05), and rno-miR-674-3p (P < 0.01), were significantly upregulated in IGF-Exo compared with Nor-Exo (Figure 4D).

### Neuroprotective effects of IGF-Exo after SCI are associated with miR-219a-2-3p-dependent inhibition of YY1

**Figure 5:**
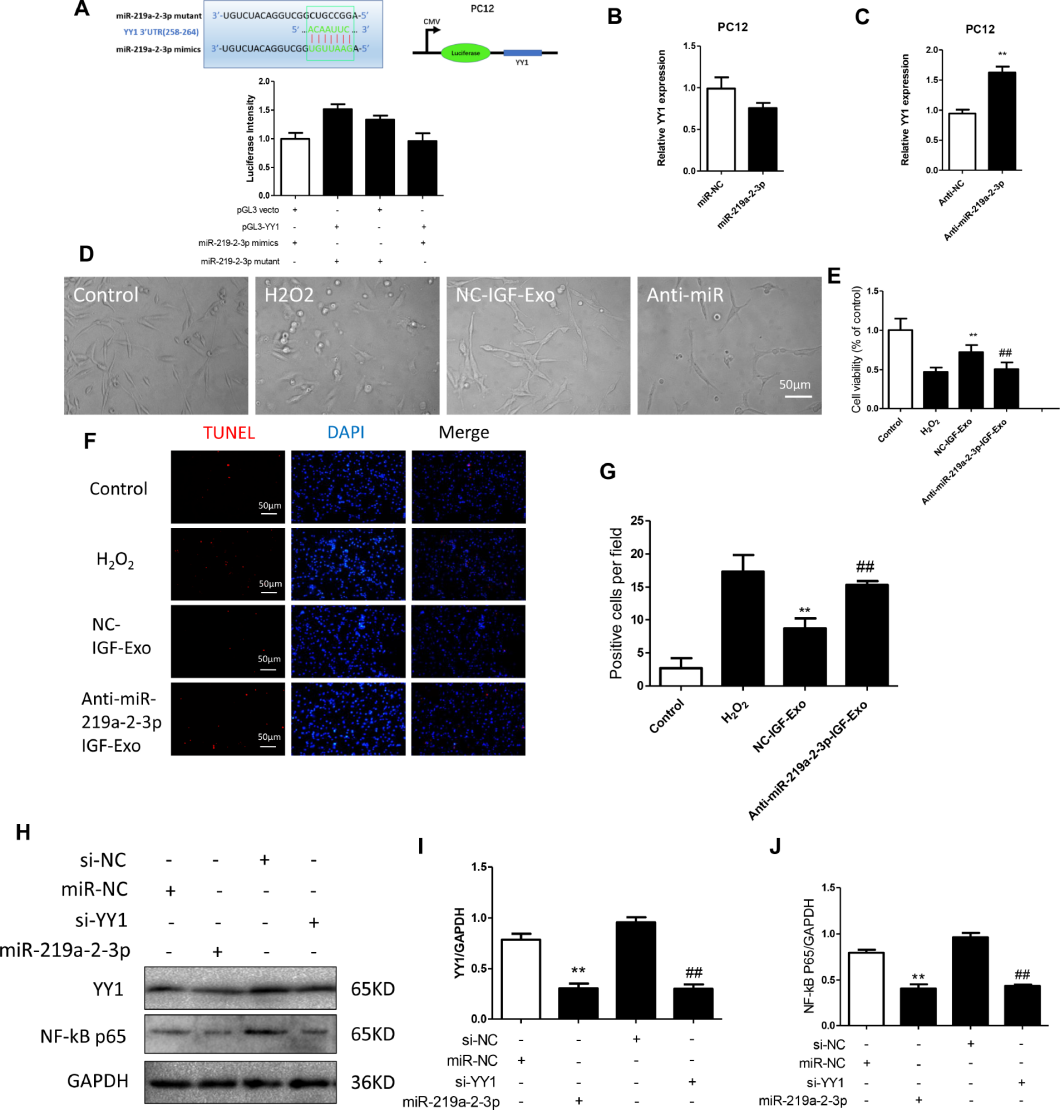
**Inhibition of miR-219a-2-3p in exosomes reduced the protective effects of IGF-Exo.** (**A**) Luciferase activity was detected in PC12 cells co-transfected with pGL3 vector, pGL3-YY1, miR-219a-2-3p mimics, or miR-219a-2-3p mutant. (**B**) Relative YY1 expression after upregulation of miR-219a-2-3p measured by qRT-PCR. B) PC12 cells were transfected with miR-219a-2-3p mimics or miR-NC; relative YY1 expression was analyzed by qRT-PCR. (**C**) PC12 cells were transfected with Anti-miR-219a-2-3p (miR-219a-2-3p inhibitor) or Anti-NC (control); relative YY1 expression was analyzed by qRT-PCR. (**D**) Morphology in each experimental group examined by light microscopy (control group: PC12 cells without treatment; H_2_O_2_ group: PC12 cells treated with H_2_O_2_ for 24h; IGF-Exo group: PC12 cells pretreated with IGF-Exo for 24h followed by H_2_O_2_ for 24h); Anti-miR-219a-2-3p-IGF-Exo group: PC12 cells pretreated with miR-219a-2-3p inhibitor-transfected IGF-Exo for 24h followed by H_2_O_2_ for 24h). (**E**) Cell viability in each experimental group when miR-219a-2-3p was downregulated in IGF-Exo; (**F**) TUNEL staining (red) indicative of apoptosis in each experimental group; DAPI in blue. (**G**) Numbers of TUNEL-positive cells per field for each experiment group. (**H**–**J**) Western Blot analysis of YY1 and NF-kB-p65 (si-NC: siRNA blank vector; miR-NC: miRNA blank vector; si-YY1: YY1 siRNA; miR-219a-2-3p: miR-219a-2-3p mimics). Data are expressed as means ± SD (analysis of variance followed by Student-Newman-Keuls *post hoc* test). **P < 0.01, *vs*. control group; ##*P* < 0.01, *vs*. SCI group.

We screened possible target genes of miR-219a-2-3p using bioinformatics methods (Target Scan v7.2) and identified YY1 was the potential target (Figure 5A). We therefore constructed a YY1-3′UTR (3′-Untranslated Region) expression vector containing a luciferase gene and transfected it into PC12 cells. A conventional luciferase reporter assay showed that miR-219a-2-3p interacted with YY1 (Figure 5A). Additionally, qRT-PCR showed that YY1 expression decreased when miR-219a-2-3p was upregulated (Figure 5B). Furthermore, YY1 expression increased when miR-219a-2-3p was downregulated (Figure 5C). Blockage of miR-219a-2-3p by Anti-miR-219a-2-3p (the miR-219a-2-3p inhibitor) inhibited IGF-Exo-mediated neuroprotective effects (Figure 5D). CCK-8 assays, TUNEL staining, and Western blots were performed to confirm that miR-219a-2-3p protected against neural injury and inhibited resulting apoptosis (Figure 5E–5G). The results indicated that miR-219a-2-3p expression downregulated, while miR-219a-2-3p knockdown upregulated, the YY1/NF-kB-p65 axis in PC12 cells (Figure 5H–5J).

## DISCUSSION

Although basic research and clinical studies have attempted to identify more effective treatments for SCI patients, the prognosis remains very poor [29]. The pathological processes involved in SCI include neuroinflammation, apoptosis, and inhibition of neural regeneration [30, 31]. A previous study revealed that exosomes derived from MSC and NSC stem cells suppress inflammation and apoptosis after neural injury. However, the mechanisms underlying these effects remain unknown. Here, we used exosomes derived from NSCs exposed to IGF-1 to treat SCI for the first time and demonstrated that these IGF-Exo enhanced the neuroprotective effects of exosomes by inhibiting apoptosis and neuroinflammation in an miR-219a-2-3p-dependent manner.

Several studies have demonstrated that stem cells (especially MSCs and NSCs) administered either systemically or via local transplantation are potential therapies for neural injury and nervous system diseases [32]. However, stem cell therapies face important challenges. Tumorigenesis due to chromosome instability and distal vascular occlusion resulting from the large size of stem cells can be fatal, and the survival rate of stem cells *in* *vivo* is relatively low [33, 34]. Fortunately, increasing evidence suggests that the biological effects of stem cells can mostly be attributed to molecules they secrete, including those secreted via exosomes [9, 35]. Recent studies indicated that exosomes derived from NSCs might aid in the discovery of treatments for central nervous system injury [36, 37], but the mechanisms underlying their protective effects are unknown, and NSCs produce relatively small quantities of exosomes.

IGF-1 improves proliferation and metabolism in neural cells, and we confirmed this IGF-1-induced increase in NSC cell viability here in a CCK-8 assay. Exosomes play an important role in intercellular communication by transmitting bioactive RNAs and proteins, and exosomes derived from NSCs have therapeutic effects against ischemia and neurodegeneration [38, 39]. Because exosomes derived from NSCs can reduce apoptosis and neuroinflammation after SCI, we hypothesized that IGF-Exo could enhance these protective effects and promote regeneration after SCI.

In this study, RNA sequencing of Nor-Exo and IGF-Exo identified 8 microRNAs that were differentially expressed between the two exosome types, and miR-219a-2-3p was determined to the most significant among them. The difference in miR-219a-2-3p expression between Nor-Exo and IGF-Exo was verified by qRT-PCR assay. Previous research indicated that miR-219a-2-3p is enriched in human white matter and oligodendrocytes, and may be a potential target in demyelinating disorders [40]. Moreover, bioinformatics screening revealed that miR-219a-2-3p interacts with the downstream gene YY1 [41]. Here, a luciferase assay confirmed that up-regulation of miR-219a-2-3p inhibited YY1 expression, while changes in YY1 expression had no effect on the expression of miR-219a-2-3p. However, YY1 expression is correlated with activity of the inflammatory NK-kB pathway, and up-regulation of YY1 enhanced inflammatory effects. Therefore, IGF-Exo likely inhibits the expression of YY1, and in turn NK-kB pathway activity, through the up-regulation of miR-219a-2-3p, thus inhibiting neuroinflammation and promoting neuroprotective effects. This mechanism was confirmed in a rescue experiment in which miR-219a-2-3p expression was inhibited by transfection of si-miR-219a-2-3p.

Some important limitations of this study should be considered when interpreting the results and should be addressed in future studies. Firstly, additional *in* *vitro* studies are needed to fully characterize the mechanism underlying the neuroprotective effects of IGF-Exo, and their efficacy should be confirmed in clinical trials. Secondly, IGF-Exo might exert neuroprotective effects and inhibit the apoptosis and inflammation via the action of other miRNAs or pathways, and additional downstream genes of miR-219a-2-3p should be examined further. Finally, additional studies are needed to verify that the effects of miR-219a-2-3p on cell apoptosis and neural regeneration observed here are applicable in other *in* *vitro* cell models and under different conditions.

In summary, this study demonstrated that exosomes derived from neural stem cells exposed to IGF-1, at least partly, suppress the nerve inflammation, inhibit apoptosis, and promote nerve regeneration via an miR-219a-2-3p-dependent mechanism after the spinal cord injury. This is the first microRNA mechanism to be identified that might explain the effects of NSC-derived exosomes in SCI and may inform the development of novel treatments for spinal cord injury.

## MATERIALS AND METHODS

### Animals

Adult female Sprague-Dawley rats (180–200g) and pregnant rats (15d) were obtained from Beijing Vital River Laboratory Animal Technology Co., Ltd (Beijing, China). All animal care and experimental protocols were approved by the Ethics Committee of the Characteristic Medical Center of Chinese People’s Armed Police Force. All animals were housed in individual cages on a 12h light/dark cycle with ad-libitum access to food and water.

### Isolation and culture of rat embryonic NSCs

15-day embryos were isolated from pregnant rats euthanized with 230 mg/kg sodium pentobarbital. Cerebral cortex tissues were then isolated from the embryos and placed in Hank’s solution. After the meninges were removed, the cerebral cortex was washed twice with Hank’s solution, cut into pieces, digested with accutase cell detachment solution at 37°C for 10 min, and dispersed by pipetting. The suspension was filtered with a 200-mesh screen and then centrifuged at 800 rpm for 5 min; NSCs were obtained from the pelleted material. The cells were then re-suspended in complete medium (DMEM/F12 medium supplemented with 20 ng/ml EGF, 10 ng/ml bFGF, 1 × B27 supplement, 100 U/mL streptomycin, and 100 U/mL penicillin). After cell density was adjusted to 1 × 10^5^/mL, cells were seeded into culture flasks and incubated at 37°C with 5% CO_2_. After 3-5 days, cells were passaged. All NSCs used in this study were from passage 3.

### Isolation and purification of NSC-Exo

Exosomes were isolated from NSC supernatant as previously described [42]. The medium was then collected and centrifuged at 300 × g for 10 min, followed by centrifugation at 2000 × g for 20 min at 4°C. After centrifugation, the supernatant was filtered using a 0.22 μm filter to remove dead cells and large cellular debris. Small cellular debris were then pelleted by centrifugation at 10,000 × g for 30 minutes, and the supernatants were recentrifuged at 100,000 × g for 70 minutes. Finally, pelleted exosomes were resuspended in 100 μL PBS solution in the ultracentrifuge tubes. Exosomes were quantitated by measuring total protein concentration using a bicinchoninic acid assay (BCA; Thermo Fisher).

### Identification of NSC-Exo

The morphology of NSC-derived exosomes (NSC-Exo) was assessed by transmission electron microscopy (TEM, Hitachi HT7700, Tokyo, Japan). Nanosizer^TM^ technology (Malvern Instruments, Malvern, UK) was used to analyze the diameter distribution of NSC-Exo. Western blotting was used to examine the specific surface markers encapsulated in the exosomes, including CD9, CD63, and Alix.

### *In vitro* experiment design

In the first *in vitro* experiment, PC12 cells were divided among the following four groups to verify the protective effect of IGF-Exo: control group (untreated PC12 cells), H_2_O_2_ group (PC12 cells treated with H_2_O_2_ for 24h), Nor**-**Exo group (PC12 cells pretreated with Nor-Exo for 24h followed by H_2_O_2_ for 24h), and the IGF**-**Exo group (PC12 cells pretreated with IGF-Exo for 24h followed by H_2_O_2_ for 24h).

In the second *in vitro* experiment, PC12 cells were divided among the following four groups to verify the effects of miR-219a-2-3p: control group (untreated PC12 cells), H_2_O_2_ group (PC12 cells treated with H_2_O_2_ for 24h), NC**-**IGF**-**Exo group (PC12 cells pretreated with NC-IGF-Exo derived from IGF-1-induced NSCs transfected with miR inhibitor negative control (Anti-NC) for 24h followed by H_2_O_2_ for 24h), and Anti**-**miR**-**219a**-**2**-**3p**-**IGF**-**Exo group (PC12 cells pretreated with IGF-Exo derived from IGF-1-induced NSCs transfected with miR-219a-2-3p inhibitor (Anti- miR-219a-2-3p) for 24h followed by H_2_O_2_ for 24h).

### *In vivo* experiment design

Adult female Sprague-Dawley rats were divided among the following four groups (n=8 per group): control group (spinal cord segment T10 was only exposed in a sham operation), SCI group (acute SCI via modified Allen’s weight drop apparatus, 0.5 mL PBS systemic administration through tail vein injection after SCI), Nor**-**Exo group (100 μg of precipitated normal exosome protein in 0.5 mL of PBS was administered via tail vein injection after SCI), and IGF**-**Exo group (100 μg of precipitated IGF-1-induced exosome protein in 0.5 mL of PBS was administered via tail vein injection after SCI).

### Cell viability assay

The viability of PC12 cells and primary neurons was evaluated with a CCK-8 assay (Dojindo, Kumamoto, Japan) to examine the effects of NSC-Exo on neural cell proliferation. The cells were rinsed three times with 1 × PBS after 0, 24, 48, and 72 h of incubation. 10 μL of CCK-8 solution diluted in 90 μL of fresh culture medium was then added followed by incubated for another 2 h at 37°C.

### Quantitative real-time RT-PCR

Total RNA was isolated from cells and tissues using Trizol reagent (TransGen Biotech, Beijing) according to the manufacturer’s instructions. For RNase R treatment, 2 mg total RNA was incubated for 15 min at 37°C with or without 3 U/mg RNase R (Epicentre Technologies, Madison, WI, USA). qRT-PCR was performed using SYBR Green (TransGen Biotech) according to the manufacturer's instructions (Table 1) and an ABI 7500 real-time PCR instrument (Applied Biosystems, Foster City, CA, USA).

**Table 1:** Primers of miRNA used for qPCR in this study.

**Primer**	**Sequence(5′to3′)**
miR-138-5p	Forward: ACACTCCAGCTGGGAGCtGGtGttGTGAATC
	Reverse: TGGTGTCGTGGAGTCG
miR-219-2-3p	Forward: ACACTCCAGCTGGGAGAATTGTGGCTGGAC
	Reverse: TGGTGTCGTGGAGTCG
miR-25	Forward: ACACTCCAGCTGGGAGGCGGAGACACGGGC
	Reverse: TGGTGTCGTGGAGTCG
miR-674-3p	Forward: ACACTCCAGCTGGGCACAGCtCCCATCTCA
	Reverse: TGGTGTCGTGGAGTCG
miR-92b	Forward: ACACTCCAGCTGGGAGGGACGGGACGCGGTGC
	Reverse: TGGTGTCGTGGAGTCG
miR-92b-3p	Forward: ACACTCCAGCTGGGTATTGCACtCGTCCCG
	Reverse: TGGTGTCGTGGAGTCG
YY1	Forward: AGTGGGAACAGAAGCAGGTG
	Reverse: GAGGTCAATGCCAGGTATCC

### Transfection

Rat miR-219a-2-3p mimics, anti-miR-219a-2-3p (miR-219a-2-3p inhibitor), si-YY1 (si-YY1), miR mimics negative control (miR-NC), miR inhibitor negative control (miR-NC), and si-YY1 negative control (si-NC) were purchased from GenePharma (Shanghai, China). Sequences are listed in Table 2.

**Table 2:** miRNA mimics, inhibitors, and siRNAs used in this study.

**Name**	**Sequence(5′to3′)**
miR-219a-2-3p mimics	AGAAUUGUGGCUGGACAUCUGU
miR-219a-2-3p inhibitor (Anti-miR-219a-2-3p)	ACAGAUGUCCAGCCACAAUUCU
siRNA-YY1(si-YY1)	GGUAAUAAGAAGUGGGAACTT
miR mimics negative control (miR-NC)	UUGUACUACACAAAAGUACUG
miR inhibitor negative control (Anti-NC)	CAGUACUUUUGUGUAGUACAA
si-YY1 negative control(si-NC)	UUCUCCGAACGUGUCACGUTT

### Cell apoptosis assays

PC12 cells were incubated with or without NSC-Exo (100 μg/mL) after treatment with H_2_O_2_ (200 μM) for 24 h. A terminal deoxynucleotidyl transferase-mediated dUTP nick end labeling assay (TUNEL; Roche, Basel, Switzerland) was used to detect DNA strand breaks according to the manufacturer’s instructions. Images were captured using a fluorescence microscope (Leica, Solms, Germany). Apoptotic neuronal cells were determined by counting total numbers of TUNEL- and DAPI-stained cells.

### Western blot analysis

Protein lysates were extracted from cells and 5 mm blocks of injured spinal cord tissue encompassing the lesion collected on days 3 and 28 after SCI by incubating with RIPA lysis and extraction buffer (KeyGEN Biotechnology, China). Protein concentration was determined using BCA. Equal amounts of proteins were separated by sodium dodecyl sulfate polyacrylamide gel electrophoresis, transferred to polyvinylidene difluoride membranes (EMD Millipore Corp., USA), incubated overnight at 4°C with primary antibodies, and blocked with bovine serum albumin (5%, v/v). The primary antibodies included Bax (1:2000, rabbit IgG; Abcam, USA), Bcl-2 (1:1000, rabbit IgG; Abcam, USA), Beclin-1 (1:1000, rabbit IgG; Abcam, USA), caspase-3 (1:1000, rabbit IgG; Abcam, USA), GAPDH (1:1000, Abcam, USA), YY1 (1:1000, mouse IgG1; Abcam, USA), and p65 (1:1000, rabbit IgG; Abcam, USA). The membranes were then incubated with secondary antibody (1:2000, Proteintech, USA) for 120 min at room temperature. Protein bands were visualized using enhanced chemiluminescence reagent (Beyotime Institute of Biotechnology, Nanjing, China), and band density was analyzed semi-quantitatively using Image J software.

### Immunofluorescence staining

For immunofluorescence staining, 1.0 x 10^5^ NSCs were cultured in 0.5 mL media unless otherwise specified. Cultured cells were fixed with 4% (wt/vol) paraformaldehyde for 30 min at room temperature, washed with 0.1 M Tris-buffered solution (pH 7.5, TBS), blocked with 10% (vol/vol) normal goat serum in TBS containing 0.3% (vol/vol) Triton X-100 at room temperature for 60 min, and incubated with primary antibodies at 4°C overnight. The primary antibodies were rabbit anti-Nestin (1:1000), rabbit anti-BrdU (1:2000), and donkey anti-NeuN (1:1000). The cells were washed with TBS and incubated with Alexa Fluor 488- and 555-conjugated secondary antibodies (1:1000, Proteintech) at room temperature for 60 min. To visualize nuclei, the cells were counterstained with 2 μg/mL 4',6-diamidino-2-phenylindole (DAPI). Finally, the cells were mounted with 80% (vol/vol) glycerol, visualized under a fluorescent microscope (IX81, Olympus Corp., Tokyo, Japan), and image data were processed using MetaMorph (Molecular Devices, Sunnyvale, CA, USA) for quantification. Numbers of positive cells were counted in three to five fandom fields per well for three separate wells in each individual experiment.

### Behavioral tests

Hindlimb motor function assessments were performed 1, 3, 7, 14, and 28 days after SCI using the Basso Beattie Bresnahan (BBB) locomotor rating scale and slanting board test. The BBB score ranged from 0 (complete hindlimb paralysis) to 21 (normal locomotion). Scores were assigned by assessing the motor capacity of the experimental rats, including the movement of the hindlimbs, weight-bearing, and coordination of forelimb and hindlimb movements. The rats were placed in an open field of wooden models for 4 min and scored by 2 researchers using blinded methods. The slanting board test was performed according to the Rivlin method and involved placing a rat on a rectangular wooden oblique board covered with a rubber pad; the oblique plate was rotated to measure the angle of the inclined plate. When the longitudinal axis of the rat’s body was parallel to the longitudinal axis of the inclined plate, the rat was tilted toward the side of the tilted plate to raise it in 5° increments. The maximum angle at which a rat could maintain its position for 5 s was measured. Measurements were performed five times for each animal, and the average values were analyzed.

### Statistical analysis

The data are presented as means ± standard deviation (SD) and were analyzed using one-way analysis of variance and Tukey’s *post* *hoc* multiple comparison tests. All experimental data were analyzed using SPSS 17.0. *P-*values < 0.05 were considered statistically significant. Additional experimental materials and methods details, including electrophysiological nerve and MRI assessments, are provided in the Supplementary Methods.

## Supplementary Material

Supplementary Methods

Supplementary Figures
