# Supplementary material for: Insulin-like growth factor-1 enhances neuroprotective effects of neural stem cell exosomes after spinal cord injury via an miR-219a-2-3p/YY1 mechanism
Source: Aging (Albany NY). 2019 Dec 17;11(24):12278–94. doi: 10.18632/aging.102568 (PMC6949049; doi:10.18632/aging.102568)
Supplement: Supplementary Methods [file aging-11-102568-s001..pdf]

SUPPLEMENTARY MATERIALS

Cells and cell culture

PC12 cells were provided by the Riken Cell Bank (Tsukuba Science City, Japan), PC12 cells were maintained in RAPI 1640 medium, containing 10% FBS, 100 IU/mL penicillin, and 100 mg/mL streptomycin. All the medium was changed every 3 days, cells were passaged every 3–5 days, according to an established protocol, 32 primary neurons were collected from embryonic (E16–18) Sprague-Dawley (SD) rats. Briefly, cerebral cortices were isolated and dissociated with trypsin (0.25%, w/v; Thermo Fisher Scientific, USA) for 20 min. Neurons were seeded at a density of 5 × 104/mL for immunofluorescent staining in 24-well culture plates and 1 × 106/mL for western blot assays in 6-well culture plates. Neurons were maintained in fresh neurobasal medium (Thermo Fisher Scientific) containing 2% B27 (2%, w/v; Thermo Fisher Scientific), 1% glutamine (Thermo Fisher Scientific), 100 IU/mL penicillin, and 100 mg/mL streptomycin. Half of the medium was changed every third day. After 5 days of cell culture, the obtained neurons were examined by microtubule.

Luciferase reporter assay

Latent binding sites were predicted by the Target Scan database (http://www.targetscan.org). PC12 cells (1 × 105 cells/well) were added to a 24-well plate and co-transfected with 200 ng pGL3-LUC-YY1 or pGL3-LUC-control vector and 80 nmol miRNA mimics or miRNA mutant mimics. After 48 h of transfection, the cells were harvested, and luciferase activities were measured with the Dual-Luciferase Reporter Assay System (Promega, Madison, WI, USA).

SCI model

The rats were anesthetized and injected intraperitoneally with chloral hydrate (300 mg/kg). The incision area was sterilized with 75% alcohol, an incision was made, and the skin was separated, then a laminectomy was performed to expose spinal cord segment T10. The impactor (weighing 10 g, 3 mm in diameter, and 200 mm in length) was obtained from the Affiliated Hospital of the Logistics University of the Chinese People's Armed Police Force. The impactor was dropped from a height of 50 mm to the surface of the spinal cord. Successfully induced SCI resulted in spinal cord congestion, tail swing reflexes, swaying legs, and slow paralysis. The wound was sutured after the spinal cord was hit. All rats were kept in a separate environment at 24°C to ensure adequate water, food, and clean bedding. The rats were provided intermittently with assisted urination 3 times daily.

MRI

While the animals were under general anesthesia, MRI scans and DTI constructions were performed by using the Siemens MAGNETOM Verso 3.0T MRI system. Since vitamin E is a fat-soluble substance that elicits high signals during MRI scanning and DTI construction, we used a vitamin E capsule as a simple marker to assist DTI to locate the scope of injury .

Nerve electrophysiological assessment

Electrophysiological assessments were including preoperative, intraoperative and postoperative measurements of the MEP for the limbs using an evoked potential (EP) instrument Viking Quest (Thermo Nicolet Corporation, USA). For preoperative measurement, amplitude and latency of the MEP were assessed: amplitude was the peak-to-peak voltage, meaning the potential difference between the most positive and the most negative peaks, and latency was time between the start of the stimulus to the appearance of the initial response wave.
