# Supplementary material for: Insulin-like growth factor-1 enhances neuroprotective effects of neural stem cell exosomes after spinal cord injury via an miR-219a-2-3p/YY1 mechanism
Source: Aging (Albany NY). 2019 Dec 17;11(24):12278–94. doi: 10.18632/aging.102568 (PMC6949049; doi:10.18632/aging.102568)
Supplement: Supplementary Figures [file aging-11-102568-s002..pdf]

SUPPLEMENTARY FIGURES

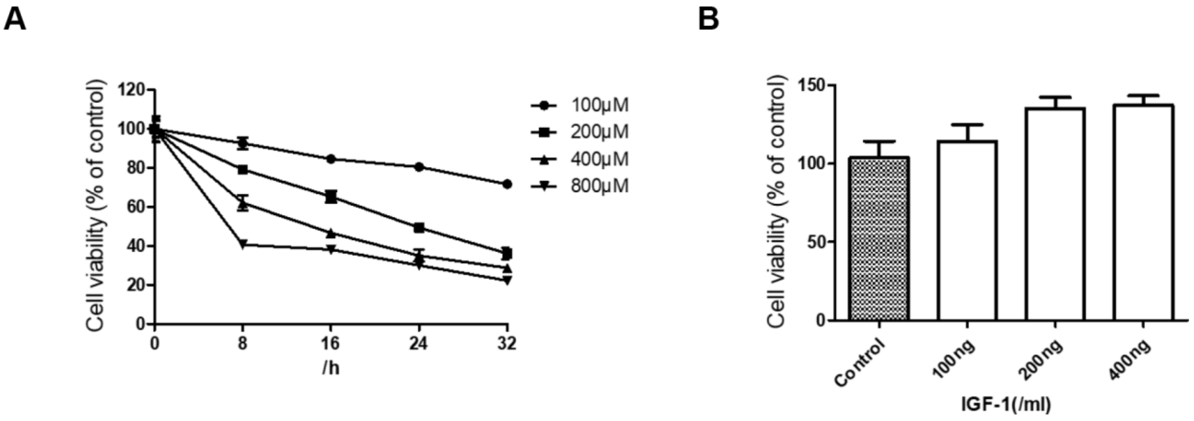

Supplementary Figure 1. IGF-Exo reduces apoptosis and promotes regeneration in neurons *in vitro*. (**A**) The CCK-8 assay of cell viability of different dose and different time of H_2O_2 on PC12 cells; (**B**) The viability of NSCs that different concentration of IGF-1 treatment.

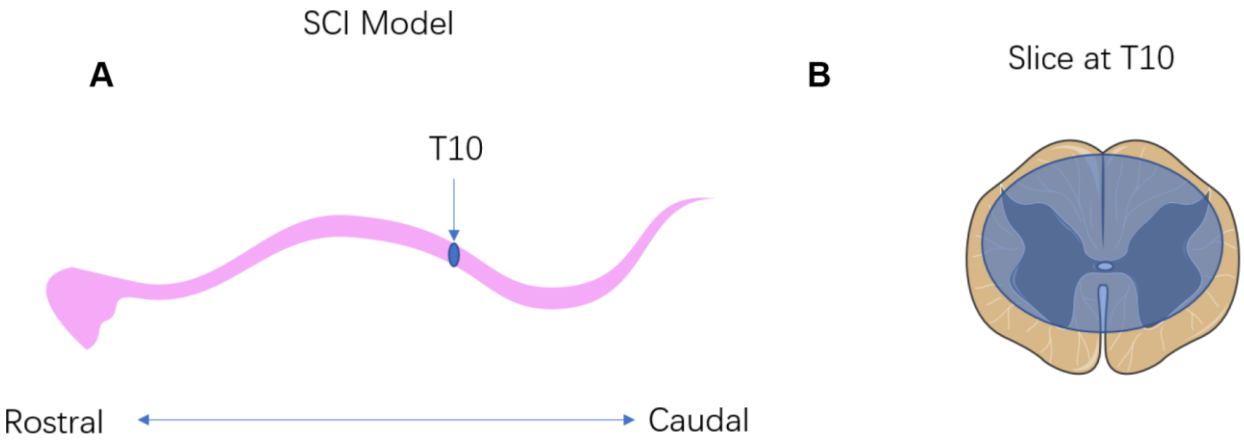

Supplementary Figure 2. Schematic of the SCI lesion area. (**A**) T10 (blue area) was crushed by modified Allen's weight drop apparatus; (**B**) The cross section of T10, blue area was injured.

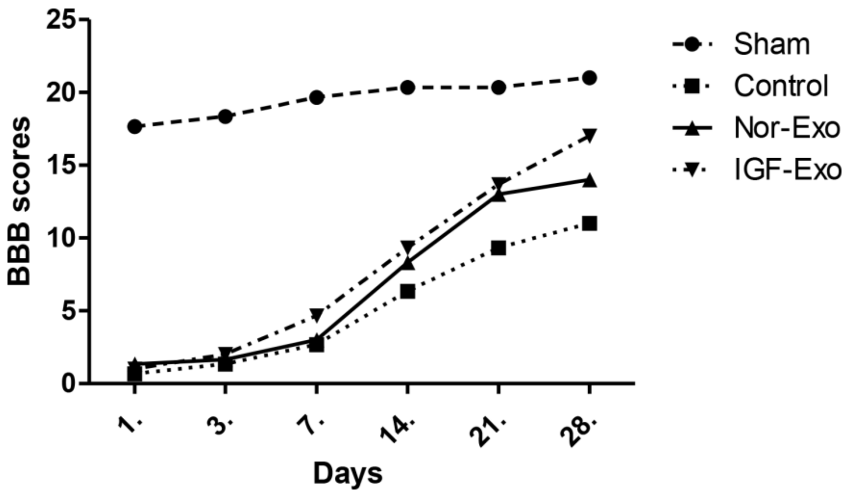

Supplementary Figure 3. BBB scores were used to assess locomotor behavior in rats 1, 3, 7, 14, and 28 days after SCI. The behavior function by BBB score at 1, 3, 7, 14 and 28 days after SCI.

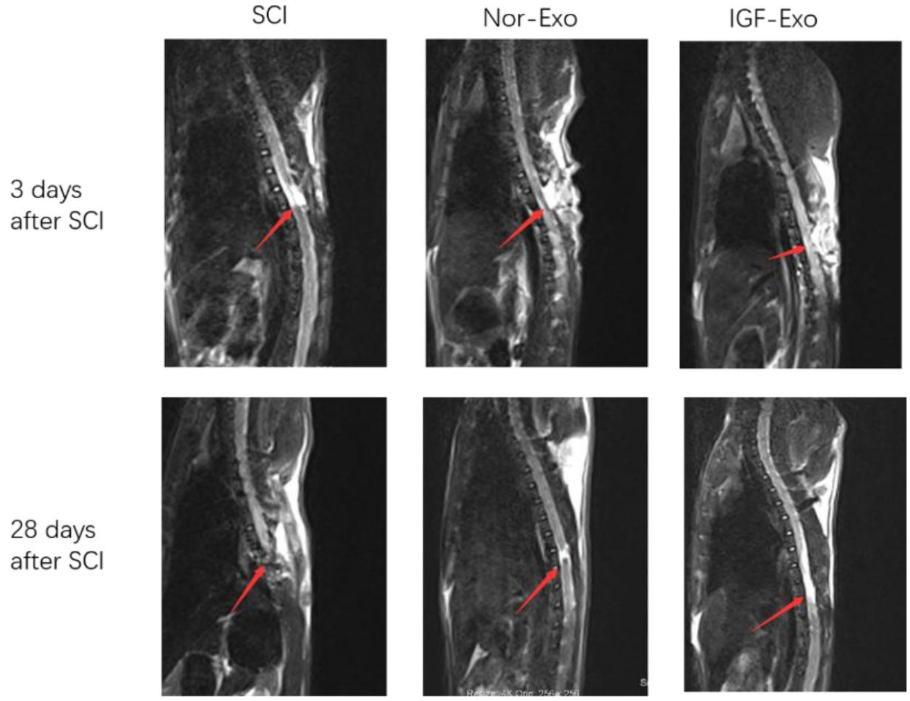

Supplementary Figure 4. The MRI image of SCI, Nor-Exo, and IGF-Exo groups. The T2 sequence of MRI at 3 days after SCI and 28 days after SCI. The red arrows point to the injury areas.

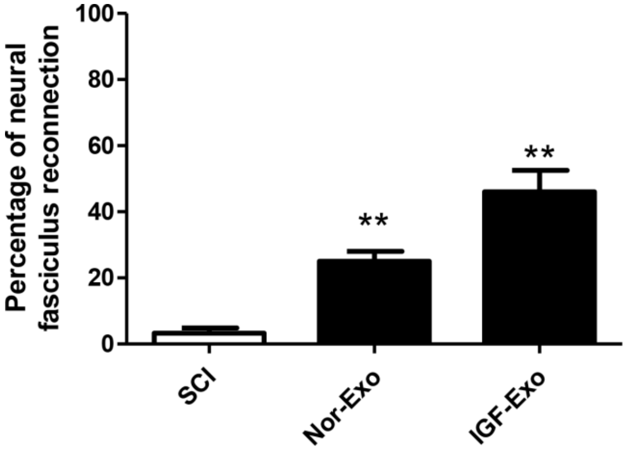

Supplementary Figure 5. Neural fasciculus reconnection percentages from DTI. The DTI sequence of MRI at 28 days after SCI showed the percentage of neural fasciculus reconnection of SCI, Nor-Exo, and IGF-Exo group. ***P*<0.01 for IGF-Exo vs. Nor-Exo.

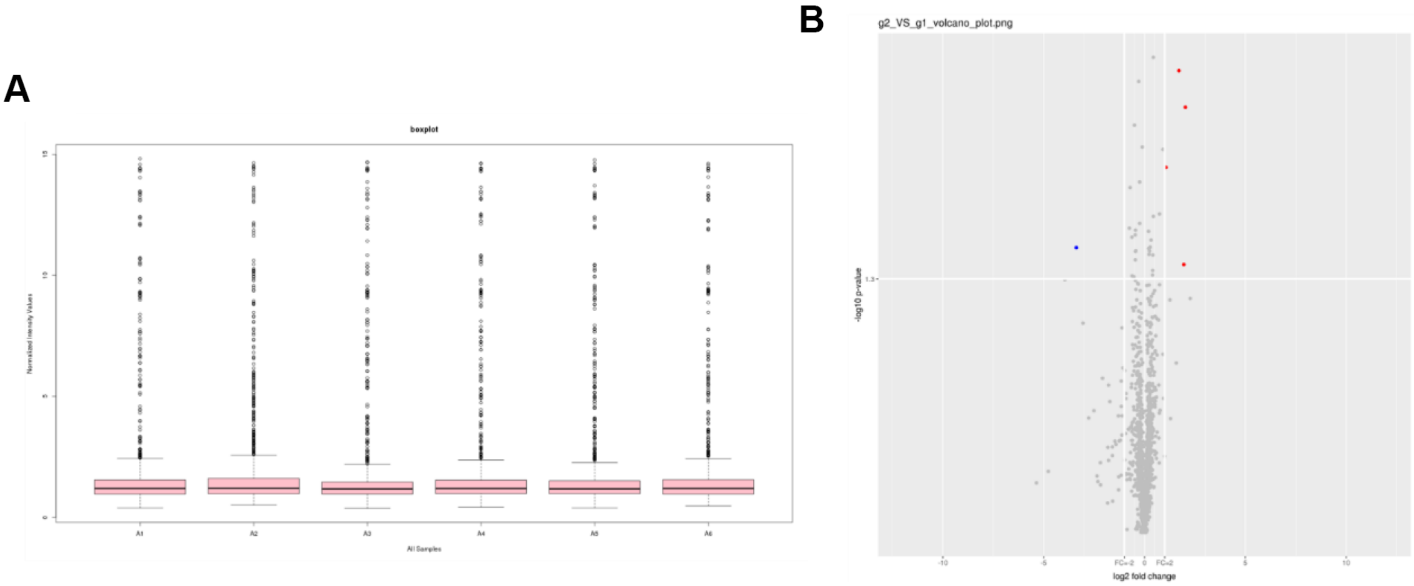

Supplementary Figure 6. miRNA sequence analysis of Nor-Exo and IGF-Exo. (**A**) all of the miRNA sequence results were subjected to normalized. (**B**) Volcano figure of miRNA sequence.
